# Correction to: Yes, they can: polar bears *Ursus maritimus* successfully hunt Svalbard reindeer *Rangifer tarandus platyrhynchus*

**DOI:** 10.1007/s00300-021-02966-6

**Published:** 2021-11-30

**Authors:** Lech Stempniewicz, Izabela Kulaszewicz, Jon Aars

**Affiliations:** 1 Department of Vertebrate Ecology and Zoology, Faculty of Biology, University of Gdańsk, Wita Stwosza 59, 80-308 Gdańsk, Poland; 2 Institute of Geophysics, Polish Academy of Sciences (IG PAS), Polish Polar Station Hornsund, Księcia Janusza 64, 01-452 Warsaw, Poland; 3 Fram Centre, Norwegian Polar Institute, 9296 Tromsø, Norway

## Correction to: Polar Biology (2021) 44:2199–2206 10.1007/s00300-021-02954-w

In the original publication of the article, the caption of the Fig. 3 was published incorrectly.

**Fig. 3** Polar bear *Ursus maritimus* with a killed reindeer *Rangifer tarandus platyrhynchus* on skerries (**a**), dragging it on to the beach (**b**), eating it (**c**) and trying to bury it (**d**). Photos: P. Klicz and P. Nowosad

**Figure:**
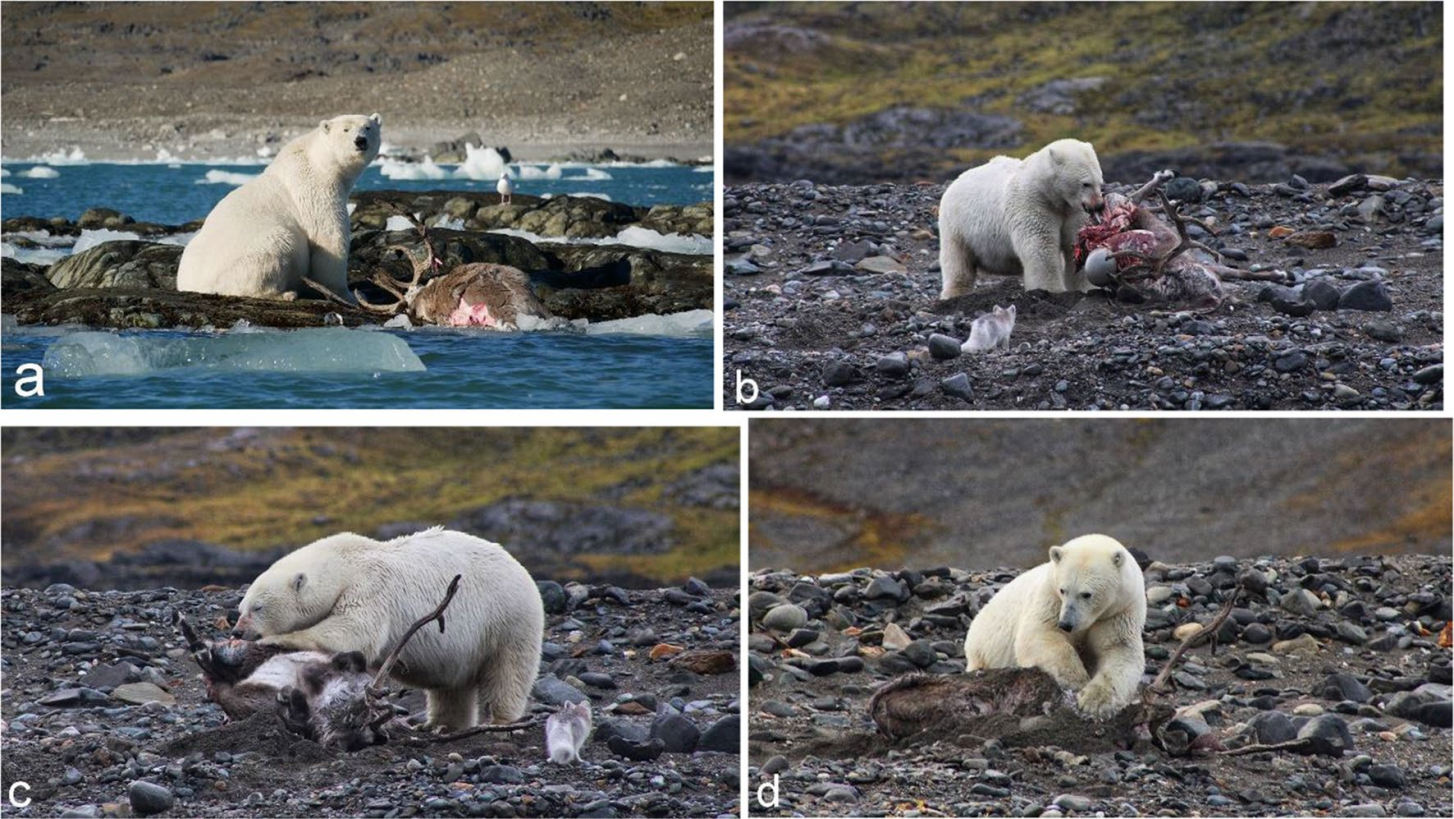

The correct caption is given in this correction.

The original article has been corrected.

